# Machine learning-assisted construction of COPD self-evaluation questionnaire (COPD-EQ): a national multicentre study in China

**DOI:** 10.7189/jogh.15.04052

**Published:** 2025-01-03

**Authors:** Yiming Ma, Zijie Zhan, Yahong Chen, Jing Zhang, Wen Li, Zhiyi He, Jungang Xie, Haijin Zhao, Anping Xu, Kun Peng, Gang Wang, Qingping Zeng, Ting Yang, Yan Chen, Chen Wang

**Affiliations:** 1 Department of Pulmonary and Critical Care Medicine, the Second Xiangya Hospital, Central South University, Changsha, China; 2 Research Unit of Respiratory Disease, Central South University, Changsha, China; 3 Clinical Medical Research Center for Pulmonary and Critical Care Medicine in Hunan Province, Changsha, China; 4 Diagnosis and Treatment Center of Respiratory Disease in Hunan Province, Changsha, China; 5 Department of Radiology, Changde Hospital, Xiangya School of Medicine, Central South University (The First People’s Hospital of Changde City), Changde, China; 6 Department of Respiratory and Critical Care Medicine, Third Hospital of Peking University, Beijing, China; 7 Department of Respiratory and Critical Care Medicine, Zhongshan Hospital of Fudan University, Shanghai, China; 8 Department of Respiratory and Critical Care Medicine, Second Affiliated Hospital of Zhejiang University School of Medicine, Hangzhou, China; 9 Department of Respiratory and Critical Care Medicine, First Affiliated Hospital of Guangxi Medical University, Nanning, China; 10 Department of Respiratory and Critical Care Medicine, Tongji Hospital, Tongji Medical College, Huazhong University of Science and Technology, Wuhan, China; 11 Department of Respiratory and Critical Care Medicine, Nanfang Hospital, Southern Medical University, Guangzhou, China; 12 Department of Respiratory and Critical Care Medicine, Yingcheng People’s Hospital, Yingcheng, China; 13 Department of Respiratory and Critical Care Medicine, Sixth Hospital of Beijing, Beijing, China; 14 Department of Respiratory and Critical Care Medicine, Anji People’s Hospital, Huzhou, China; 15 Department of Intensive Care Unit, Longshan People’s Hospital, Xiangxi, China; 16 Department of Pulmonary and Critical Care Medicine, Center of Respiratory Medicine, China-Japan Friendship Hospital, Beijing, China; 17 Institute of Respiratory Medicine, Chinese Academy of Medical Sciences, Beijing, China; 18 National Clinical Research Center for Respiratory Disease, Beijing, China; 19 National Center for Respiratory Medicine, Beijing, China; 20 Chinese Alliance for Respiratory Diseases in Primary Care, Beijing, China; 21 Chinese Academy of Medical Sciences, Peking Union Medical College, Beijing, China; 22 Department of Respiratory Medicine, Capital Medical University, Beijing, China

## Abstract

**Background:**

Approximately 70% of chronic obstructive pulmonary disease (COPD) is underdiagnosed worldwide. We aimed to develop and validate a COPD self-evaluation questionnaire (COPD-EQ) that is better suited for COPD screening in China.

**Methods:**

We developed a primary version of COPD-EQ based on the Delphi method. Then, we conducted a nationwide multicentre prospective to validate our novel COPD-EQ screening ability. To improve the screening ability of COPD-EQ, we used a series of machine learning (ML)-based methods, including logistic regression, XgBoost, LightGBM, and CatBoost. These models were developed and then evaluated on a random 3:1 train/test split.

**Results:**

Through the Delphi approach, we developed the primary version of COPD-EQ with nine items. In the following prospective multicentre study, we recruited 1824 outpatients from 12 sites, of whom 404 (22.1%) were diagnosed with COPD. After the score assignment assisted by ML models and the Shapley Additive Explanation method, six of nine items were retained for a briefer version of COPD-EQ. The scoring-based method achieves an AUC score of 0.734 at a threshold of 4.0. Finally, a novel six-item COPD-EQ questionnaire was developed.

**Conclusions:**

The COPD-EQ questionnaire was validated to be reliable and accurate in COPD screening for the Chinese population. The ML model can further improve the questionnaire’s screening ability.

Chronic obstructive pulmonary disease (COPD) is a major cause of morbidity and mortality around the world, leading to substantial economic and social burden [1]. The latest global systematic review demonstrated that the prevalence of COPD among people aged 30–79 years was 10.3%, translating to 391.9 million people around the world [2]. A national epidemiological study estimated that the overall prevalence of spirometry-defined COPD in Chinese adults was 8.6% and that there are 99.9 million COPD patients in China [3]. The substantial increase in COPD patients remains an ongoing challenge given the rapidly ageing Chinese population [4].

Given the substantial burden of COPD, early identification and diagnosis are crucial for effective management and prevention of disease progression. However, the uptake of pulmonary function tests, the gold standard for COPD diagnosis, remains low due to equipment, only 9.7% of the population had ever received a pulmonary function test in China Pulmonary Health study [3]. Previous estimates suggested that approximately 70% of COPD were underdiagnosed worldwide, and that factors including lack of spirometry, sex, age, and smoking status were associated with underdiagnosis [5]. Thus, varying degrees of diagnostic delay exist in patients with COPD, which may significantly trigger the disease progression of COPD [6]. In this context, screening questionnaires offer a practical alternative; in primary care, for example, applying them before pulmonary function test was shown to be cost-effective in identifying undiagnosed symptomatic COPD patients at high risk [7–9].

Currently, many questionnaires have been established and validated in screening COPD patients, such as COPD Population Screening Questionnaire (COPD-PS) [10], COPD Screening Questionnaire (COPD-SQ) [11], COPD Diagnostic Questionnaire (CDQ) [12], Lung Function Questionnaire (LFQ) [13], COPD Assessment in Primary Care to Identify Undiagnosed Respiratory Disease and Exacerbation Risk Questionnaire (CAPTURE) [14], and Salzburg COPD Screening Questionnaire (SCSQ) [15]. However, significant variations exist among them regarding their items and performance. Most questionnaires included the item ‘smoking,’ an essential environmental risk factor associated with COPD, as well as the item ‘old age’. Nevertheless, there is a lack of a uniform screening standard for items evaluating symptoms related to COPD. Bastidas et al. [16] reported varied discriminatory capacities in clinical practice when comparing five diagnostic questionnaires for COPD, with the area under the receiver operating characteristics curve ranging from 0.581 to 0.681. Considering the above analysis, we now propose a novel COPD self-evaluation questionnaire (COPD-EQ) which is better suited for COPD screening in China. 

## METHODS

### Study design

We first drafted a nine-item COPD-EQ questionnaire following a three-stage Delphi method. Then, we designed a nationwide multicentre study to validate and refine the questionnaire. To make the COPD-EQ questionnaire convenient to use, we determined the optimal score assignments of items under the guidance of machine learning (ML) algorithms and SHapley Additive exPlanation (SHAP) method [17]. The final score assignment scheme showed that the drafted COPD-EQ questionnaire can be shortened to six questions. Compared with COPD-PS, the scored COPD-EQ can achieve significantly higher screening precisions with a lower false negative rate. Considering recent studies that showed ML models can help to improve the performances of respiratory disease monitoring and diagnosis [18], we also constructed ML models by treating the answers of the questionnaire items as features. Experiments with the data collected in the study show that ML models can significantly improve screening performances of the COPD-EQ questionnaire.

### Study phases

The study consists of three phases: the development of the primary COPD-EQ questionnaire; its validation and optimisation; and the use of ML models to optimise its diagnostic performance.

#### Phase I: Development of the primary COPD-EQ questionnaire

We developed a COPD-EQ questionnaire based on the Delphi process, which is commonly used in health care to develop consensus among a group of experts. We began this stage by constructing a pool of candidate items that were associated with risk factors of COPD, inclusive of items in previously reported COPD screening questionnaires. Next, we constructed an expert consultation questionnaire (COPD-EQ I) and administered it to an expert panel, which comprised 13 pulmonologists from the Chinese Medical Association working in the field of COPD, gathered through WeChat or e-mail calls for the first round of the survey. The questionnaire consists a description of the questionnaire, a section collecting the experts' demographic data, an evaluation of the importance of items in the pool based on the five-point Likert scale, an evaluation of experts’ familiarity, and a self-judgment of their knowledge and competence and suggestion or questions. In the second round, we developed the consultation questionnaire (COPD-EQ II) based on the results of COPD-EQ I. Some items were dropped in COPD-EQ II based on the coefficient of variance (CV) and the mean value of importance. We then administered this new questionnaire to experts in an expert meeting and obtained their consensus on its content, leading to the development of the primary version of the COPD-EQ.

#### Phase II: Data collection

To further refine the COPD-EQ and identify the most predictive items of airflow obstruction (post-bronchodilator FEV1/FVC<0.7), we tested the primary COPD-EQ questionnaire between June 2021 and December 2021 among outpatients from 12 hospitals nationwide, including eight tertiary hospitals and four secondary hospitals. We enrolled subjects aged between 35 and 80 years old who agreed to participate in the study and signed an informed consent document. Subjects were excluded if they had been diagnosed with chronic pulmonary diseases, such as asthma, tuberculosis, interstitial lung disease, lung cancer, bronchiectasis and COPD; had a history of lung resection; had an allergy to bronchodilators; had participated in other clinical studies; visited a hospital for treatment due to acute exacerbation of pulmonary diseases; had disturbance of consciousness, language disorders, or comprehension impediment; were not able to complete pulmonary function test; lacked or missed core data, including data on pulmonary spirometry or responses in primary COPD-EQ questionnaire and COPD-PS questionnaire. We also used multiple imputation methods to handle missing data in the enrolled subjects. All subjects underwent spirometry and completed the COPD-EQ and COPD-PS questionnaires. Subjects with post-bronchodilator FEV1/FVC<0.70 were diagnosed with COPD. We also collected several characteristics of subjects at study entry, including demographic data (i.e. age, sex, race, weight, height, smoking status, smoking pack-years), history of present illness (i.e. whether failing to work longer than one day because of respiratory problem in the past four weeks, whether visiting emergency clinic or hospitalised because of respiratory problem in the past three months), comorbidities, family history of respiratory diseases, and allergic history.

#### Phase III: ML model construction and scoring optimisation

In this phase, we first construct a scoring scheme for COPD-EQ for convenient clinical usage, similar to the COPD-PS. Scoring each choice of each item in COPD-EQ is computationally intractable if a full grid search is conducted. Thus, we reduce the search space with the guidance of ML algorithms. Our final optimal scoring scheme can achieve better screening performances than COPD-PS. Considering the recent studies of ML algorithms in respiratory disease monitoring and diagnosis [18], we construct ML models by treating each item’s answers as features. The validity of the COPD-EQ and ML models constructed are evaluated in terms of area under curve (AUC) [19], sensitivity, specificity, the Youden Index, and ROC curves.

#### Feature engineering in preprocessing

Here we mainly consider the question items or their choices as the features for developing required ML models and denote a question item as ‘Q’. For binary questions, the question itself can serve as the whole feature name. For multi-choice questions, the choices are numbered with I (equal to 0 up to 4) based on their degrees of medical significance, and a feature coming from a choice item is denoted as {Q}_{i}. The second question in COPD-EQ contains a sub-query on the smoking amount, which is a numerical question whose unit is packed per year. We discretised its numerical answers into three categories according to the previous study [20]: <200 cigarettes per year; <400 cigarettes per year and ≥200 cigarettes per year; ≥400 cigarettes per year. The other sub-query in the second question was whether the patient is a smoker. If the answer was no, we transformed it into the fourth category (i.e. zero cigarettes per year).

#### SHAP-based item importance ranking and model fitting

A usual approach to ranking would be fitting a logistic regression (LR) model [21] and using the coefficients to estimate the item scoring, as described in a previous study [11]. However, this method was not applicable for our study, so we ranked the importance of question items via features’ SHA*P* values calculated on fitted ML models. The SHAP method is an additive post-hoc explanation method that generates more reliable explanations than the feature importance scores like model coefficients in an LR model. We first split the data set into an 8:2 train and test splits; we then fit the model on the train split and measured the SHA*P* values of the features on the test split. Then we obtained multiple rankings via stratified cross-validation because feature importance ranking in a single train split might be biased due to the randomness of the data set split. We conducted 5-fold stratified cross-validation, whereby we fitted a categorical boosting (CatBoost) model on each fold on the train split and calculated the SHAP-based importance scores on the test split.

#### Optimising item scoring scheme through grid search

We then considered narrowing down the search space according to the ranking results: the answer items in the top two questions had a maximum score equal to three; the answer items in the third to fifth-ranked questions had a maximum score equal to two; the answer items in the sixth and seventh-ranked questions had the maximum score equal to one. The rest of the question items were directly eliminated since their choices’ scores are all zero. For the questionnaire’s scoring scheme to make sense medically, the detailed choice item scores had to be in strict consistency with their medical severity. To ensure the practicality of the scoring scheme, we restricted the scores for each answer item to between 0.5–3 and the scores had to be an integer multiple of 0.5. With the above restriction, the size of the item scoring search space was reduced from 2.19 ^+ 16^ to 1.29 ^+ 7^. We then determined each choice item’s score via grid search. We explored all the possible combinations of score assignments and found the scoring scheme receiving the highest AUC score on our data set. Since different thresholds correspond to different AUC scores, we defined the AUC score for a scoring scheme as the best AUC score it can achieve.

#### Phase IV: Validation of validity and reliability

The discriminate validity and test-retest reliability of COPD-EQ were evaluated in this phase. To demonstrate that the answers filled out by the subjects are stable and reliable, a subsample of 96 subjects was randomly selected for validation of the COPD-EQ test-retest reliability. Those subjects were asked to complete the questionnaire at both study entry and at the two-week follow-up. Both Pearson and the intra-class correlation coefficients were evaluated. To analyse discriminate validity, the Pearson product-moment correlation coefficients between COPD-EQ score and post-bronchodilator FEV1, FEV1 percent predicted and FEV1/FVC were calculated [14].

#### Phase V: Development and comparison with additional ML models

We evaluated our model via a random 3:1 train/test split. Specifically, we implemented several widely-used ML-based models, including support vector machine (SVM), naïve Bayes (NB), random forest (RF), K-nearest neighbourhood (KNN), LR, extreme gradient boosting (XgBoost) [22], light gradient boosting machine (LightGBM) [23], and CatBoost [24]. CatBoost is an ordered gradient boosting algorithm which uses ordered target-based statistics for categorical feature processing and permutation strategies to avoid prediction shift. Its base learner is an oblivious tree, and each tree corresponds to a partition of the feature space. The model learns the feature space partition at each training iteration and finally obtains the aggregated data as a classification result. CatBoost achieves SOTA performances on a wide range of tasks [25]. Meanwhile, LR approximates the likelihood ratio via a linear regression, can be combined with LASSO or L2 regularisation, and has been widely used in the medical domain [26].

In our study, we applied a cross-validation grid-search strategy to tune the hyper-parameters on the train set for all models, including maximum tree depth; maximum iteration number; learning rate; L2 leaf regularisation and early stopping rounds for CatBoost, LightGBM, and XgBoost; margin cost parameter; kernel parameter and kernel type for SVM; penalty strength parameter; and maximum iteration number for LR.

### Statistical analysis

We summarised continuous variables as medians and interquartile ranges (IQRs) and compared them with non-parametric Wilcox’s test. We presented categorical variables as numbers and percentages and compared them using the χ^2^ test or Fisher’s exact test. A two-sided *P*-value <0.05 was considered statistically significant. We performed all statistical analyses and model constructions in R, version 4.1.0(R Core Team, Vienna, Austria) and Python, version 3.8.0 (Python Software Foundation, Wilmington, Delaware, USA).

## RESULTS

### Development of the primary COPD-EQ questionnaire

We determined nine items for the primary COPD-EQ questionnaire through the Delphi method, grouped into three categories: personal history (age, cigarette smoking index, biomass exposure, respiratory disease in childhood), family history (chronic respiratory disease in first-degree relatives), and clinical manifestations (condition for shortness of breath, frequency for shortness of breath, cough, expectoration) (**Table 1**; Table S1 in the **Online Supplementary Document**).

**Table 1:** The primary COPD-EQ questionnaire obtained by the Delphi method

Response choices by item questions	Choice id
How old are you?	
*Aged 25–49*	Item-1: age_0
*Aged 50–59*	Item-1: age_1
*Aged 60–69*	Item-1: age_2
*Aged ≥70*	Item-1: age_3
How many cigarettes have you smoked? (packs per year)	
*0*	Item-2: smoking_amount_0
*0–10*	Item-2: smoking_amount_1
*10–20*	Item-2: smoking_amount_2
*>20*	Item-2: smoking_amount_3
Have you been exposed to biomass smoke for more than half a year?	
*No*	Item-3: bio_fuel_0
*Yes*	Item-3: bio_fuel_1
During the past year, how much of time did you feel short of breath?	
*None of the time*	Item-4: short_breathe_0
*A little of the time*	Item-4: short_breathe_1
*Some of the time*	Item-4: short_breathe_2
*Most of the time*	Item-4: short_breathe_3
*All the time*	Item-4: short_breathe_4
When do you have dyspnoea?	
*No dyspnoea*	Item-5: dyspnoea_0
*During strenuous activities*	Item-5: dyspnoea_1
*During walking quickly on the flat ground or climbing a small slope*	Item-5: dyspnoea_2
*Slower than peers when walking on the flat ground, or needs to rest*	Item-5: dyspnoea_3
*Severe dyspnoea leads to inability to leave home, or dyspnoea when wearing and undressing*	Item-5: dyspnoea_4
Do you often cough when you don't have a cold?	
*No*	Item-6: cough_0
*Yes*	Item-6: cough_1
Have you ever coughed up something, such as mucus or sputum?	
*Never*	Item-7: cough_up_0
*Yes, cough occasionally when having a cold or chest infection*	Item-7: cough_up_1
*Yes, cough for a few days every month*	Item-7: cough_up_2
*Yes, cough for most days*	Item-7: cough_up_3
*Yes, cough every day*	Item-7: cough_up_4
Did you suffer from chronic respiratory disease as a child?	
*No*	Item-8: childhood_ resp_disease_0
*Yes*	Item-8: childhood_ resp_disease_1
Whether your first-degree relatives (parents, children, siblings) have a history of respiratory disease (such as chronic bronchitis, emphysema, asthma)?	
*No*	Item-9: relatives_medical_history_0
*Yes*	Item-9: relatives_medical_history_1

#### Collected data set and subject characteristics

We assessed 2105 patients for eligibility, with 1824 patients being included in the study. Among a total of 1824 subjects, 404 (22.1%) were COPD patients (**Table 2**). The average age of the subjects was 57.1 years and 38.37% were male.

**Table 2:** Characteristics of study subjects in COPD and non-COPD groups

Responses by items	COPD, n (%)	Non-COPD, n (%)	χ^2^	*P*-value
Item-1: age	404 (0)	1420 (0)	119.3	0.00
*35–49 y*	38 (9.4)	389 (27.4)		
*50–59 y*	115 (28.4)	546 (38.4)		
*60–69 y*	156 (38.6)	340 (23.9)		
*≥70 y*	95 (23.5)	145 (10.2)		
Item-2: smoking_amount	404 (4)	1420 (6)	11.08	0.00
*x̅ (SD)*	27.8 (35.7)	12.0 (21.4)		
*MD (IQR)*	20.0 (0.0–40.0)	0.0 (0.0–20.0)		
*Min–Max*	0.0–365.0	0.0–365.0		
Item-3: bio_fuel	404 (0)	1420 (0)	40.56	0.00
*No*	222 (54.9)	1020 (71.8)		
*Yes*	182 (45.0)	400 (28.2)		
Item-4: short_breathe	404 (0)	1420 (0)	140.3	0.00
*Never*	91 (22.5)	627 (44.2)		
*Few times*	75 (18.6)	331 (23.3)		
*Sometimes*	112 (27.7)	315 (22.2)		
*Often*	101 (25.0)	128 (9.0)		
*Always*	25 (6.2)	19 (1.3)		
Item-5: dyspnoea	404 (0)	1420 (0)	103.1	0.00
*No difficulty*	103 (25.5)	654 (46.1)		
*Difficult when intense exercise*	109 (27.0)	445 (31.3)		
*Difficult when climbing*	141 (34.9)	242 (17.0)		
*Difficult when walking*	48 (11.9)	75 (5.3)		
*Always difficult*	3 (0.7)	4 (0.3)		
Item-6: cough	404 (0)	1420 (0)	24.33	0.00
*No*	224 (55.4)	980 (69.0)		
*Yes*	180 (44.6)	442(31.0)		
Item-7: cough_up	404 (0)	1420 (0)	49.45	0.00
*Never*	103 (25.5)	577 (40.6)		
*Sometimes*	123 (30.4)	457 (32.2)		
*Every month*	65 (16.1)	135 (9.5)		
*Most days*	44 (10.9)	90 (6.3)		
*Everyday*	69 (17.1)	161 (11.3)		
Item-8: childhood_ resp_disease	404 (0)	1420 (0)	0.05	0.83
*No*	387 (95.8)	1357 (95.6)		
*Yes*	17 (4.2)	63 (4.4)		
Item-9: relatives_medical_history	404 (0)	1420 (0)	6.93	0.01
*No*	300 (74.3)	1139 (80.2)		
*Yes*	104 (25.7)	281 (19.8)		

#### Item score assignment

We treated each choice of the COPD-EQ questionnaire (**Table 2**) as a feature for the LR model; this model fits the data set to calculate the coefficients for each feature. The coefficients in an LR can be seen as a measurement of feature importance.

**Figure 1:**
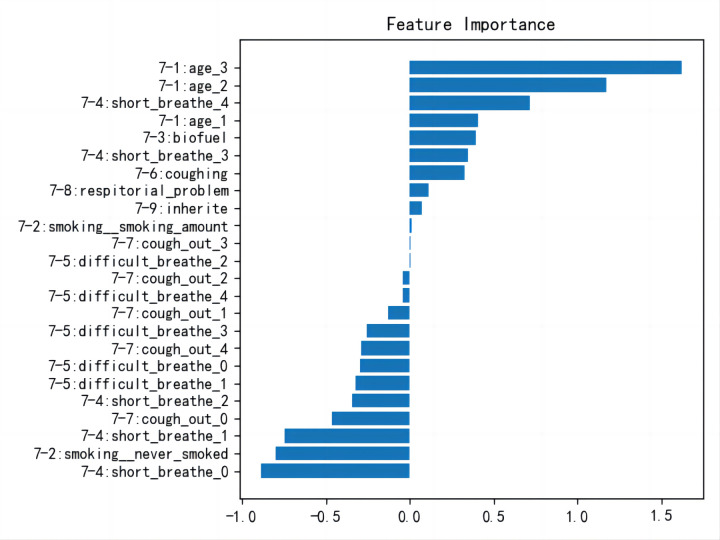
Coefficients of each choice in the COPD-EQ questionnaire analysed by logistic regression model.

The LR model’s coefficients differ in signs; this is different from the COPD-SQ [11] in which all the coefficients of their LR model are positive (**Figure 1**). Thus, we had to use the absolute values of the coefficients to evaluate the importance scores of each choice. However, ranks via the absolute coefficients in our model conflicted with medical common sense. In the above-mentioned COPD-SQ study, the response choices corresponding to the more medically severe scenarios consistently obtained larger coefficients. However, our model did not obtain similar results. For example, the response choice ‘Item-5: dyspnoea_1’ (the second choice of the question Item-5) had an absolute coefficient larger than the feature ‘Item-5: dyspnoea_3′ (the fourth choice of Item-5); However, the latter is more medically severe than the former. Similarly, the feature ‘Item-4: short_breathe_0’ (the first choice of Item-4) has an absolute coefficient larger than the feature ‘Item-4: short_breathe_1’ (the second choice of Item-4) and feature ‘Item-4: short_breathe_2’ (the third choice of Item-4). This suggested that the method in the COPD-SQ study [11] did not result in medically sound item scoring schemes for us.

We instead ranked the importance of each question item. We fit the CatBoost model on 5-folds with the answer to a question as a feature and calculated the SHA*P* values of each question item, which we then used to rank the questions (**Table 3**). These five different folds resulted in five different ranks. Thus, to obtain a reliable question item ranking, we had to ensemble all the ranks from different folds to obtain a final rank by summing all the ranks from 5-folds. Question item 1, 4, and 2 obtained the highest rank, while items 9 and 8 ranked the lowest.

**Table 3:** The SHA*P* values and ensemble ranks of the ML models on each fold of the cross-validation

	Fold 1		Fold 2		Fold 3		Fold 4		Fold 5		
**Item**	**SHAP value**	**Rank**	**SHAP value**	**Rank**	**SHAP value**	**Rank**	**SHAP value**	**Rank**	**SHAP value**	**Rank**	**Final Rank**
1	0.45	3	0.47	2	0.49	1	0.50	1	0.49	1	1
2	0.46	2	0.31	3	0.27	3	0.35	3	0.33	3	3
3	0.17	5	0.16	4	0.23	4	0.19	4	0.17	4	4
4	0.46	1	0.50	1	0.42	2	0.39	2	0.42	2	2
5	0.03	7	0.07	7	0.08	6	0.06	7	0.00	8	7
6	0.17	4	0.15	5	0.13	5	0.12	5	0.12	5	5
7	0.07	6	0.08	6	0.06	7	0.11	6	0.07	6	6
8	0.01	8	0.02	8	0.01	9	0.01	9	0.00	9	9
9	0.00	9	0.01	9	0.03	8	0.03	8	0.02	7	8

SHAP – SHapley Additive explanation

With the guidance of the question item ranking, the grid search of the detailed item scoring scheme became computationally tractable (**Table 4**). Since there were three question items (i.e. Item-6, Item-8 and Item-9) that received all zero scores, they could be discarded, leading to a final six-item version of the questionnaire (Table S2 in the **Online Supplementary Document**). In our data set, the COPD-EQ questionnaire with this scoring scheme achieved satisfactory screening performance. According to the Youden Index (equal to sensitivity plus specificity minus 1), threshold 4.0 of the total score achieved the best balance between sensitivity and specificity. We can see that COPD-EQ achieved an AUC score of 0.734, which is significantly higher than the AUC score of COPD-PS of 0.693 at threshold 3 (**Table 5**).

**Table 4:** The optimal scoring scheme obtained under the guidance of ML algorithms and the SHAP method

Items	Questions	Responses	Score
Item-1: age	How old are you?	Aged 35–49	0
		Aged 50–59	1
		Aged 60–69	1.5
		Aged ≥70	3
Item-2: smoking_amount	How many cigarettes have you smoked? (packs per year)	0	0
		0–10	1.5
		10–20	1.5
		>20	1.5
Item-3: bio_fuel	Do you have exposed to biomass smoke for more than half a year?	No	0
		Yes	0.5
Item-4: short_breathe	During the past year, how much of time did you feel short of breath?	None of the time	0
		A little of the time	0.5
		Some of the time	0.5
		Most of the time	1.5
		All the time	2
Item-5: dyspnoea	When do you have dyspnoea?	No dyspnoea	0
		During strenuous activities	0.5
		During walking quickly on the flat ground or climbing a small slope	0.5
		Slower than peers when walking on the flat ground, or needs to rest	0.5
		Severe dyspnoea leads to inability to leave home, or dyspnoea when wearing and undressing	1
Item-7: cough_up	Have you ever coughed up something, such as mucus or sputum?	Never	0
		Yes, cough occasionally when having a cold or chest infection	1
		Yes, cough for a few days every month	1
		Yes, cough for most days	1
		Yes, cough every day	1

SHAP – SHapley Additive exPlanation

**Table 5:** The comparisons of COPD-EQ and COPD-PS on the screening performances under different score thresholds*

	COPD-EQ			COPD-PS		
**Threshold**	**AUC**	**Sensitivity**	**Specificity**	**AUC**	**Sensitivity**	**Specificity**
0.0	51.18	99.75	2.60	53.51	99.01	8.00
0.5	51.29	99.00	3.58			
1.0	54.80	98.50	11.10	58.89	96.32	21.46
1.5	57.75	95.26	20.24			
2.0	60.32	91.27	29.37	65.04	85.05	45.02
2.5	64.07	86.53	41.60			
3.0	67.80	79.80	55.80	69.32	74.27	64.38
3.5	71.37	72.82	69.92			
4.0	73.39	67.58	79.20	68.51	54.41	82.61
4.5	69.27	51.62	86.93			
5.0	64.98	36.91	93.04	64.00	36.28	91.73
5.5	60.95	26.18	95.71			
6.0	57.27	17.21	97.33	61.55	25.98	97.13
6.5	54.62	10.22	99.02			
7.0	52.69	5.74	99.65	56.88	14.95	98.81
7.5	51.76	3.74	99.79			
8.0	50.87	1.75	100.00	52.40	5.15	99.65
8.5	50.00	0.00	100.00			
9.0	50.00	0.00	100.00	50.05	0.25	99.86

AUC – area under curve

*All values are expressed as percentages.

#### Discriminate validity

COPD-EQ scores were significantly associated with post bronchodilator FEV1 (r = −0.332; *P* < 0.001), post-bronchodilator FEV1 percent predicted (r = −0.324; *P* < 0.001), and post-bronchodilator FEV1/FVC (r = −0.409; *P* < 0.001).

#### Test-Retest reliability

A subgroup of 96 randomly selected participants completed COPD-EQ at both study entry and two-week follow-up. The Pearson and intra-class correlation coefficients of their responses for COPD-EQ were 0.89 in the first and 0.94 in the second round of screening. The high Pearson correlation in the test-retest reliability proved the questionnaire's validity.

#### Performances of ML models

**Figure 2:**
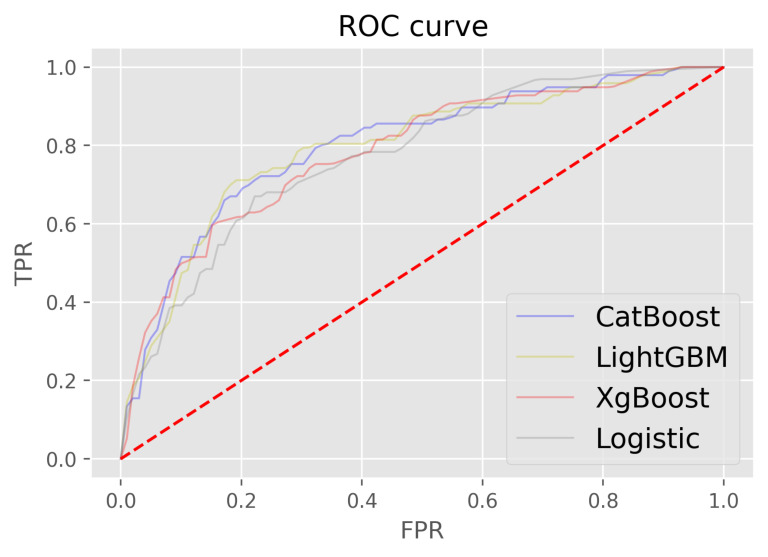
ROC curves of ML models for COPD-EQ. ROC – receiver operating characteristics, ML – machine learning, CatBoost – categorical boosting, LightGBM – light gradient boosting machine, XGBoost – extreme gradient boosting, TPR – true positive rate, FPR – false positive rate.

Treating the response of each question item as a feature allowed us to build an ML model to predict the screening result, instead of summing up all the scores obtained in the questionnaire. We fit the ML models under the diagnostic criteria of FEV1/FVC<0.7 and ran them for 10 times under different random seeds, determining their results as the means and standard deviations of their AUC scores (**Figure 2**, **Table 6**). We then selected the cut-off values to obtain the highest Youden’s index. Among all the models, CatBoost achieved the highest average AUC score on the test set. At the best Youden’s index of 0.528, the model’s sensitivity was 0.818 and the specificity was 0.711. The ML models obtained high AUC scores, showing their potential in COPD screening.

**Table 6:** The performance of ML models of COPD-EQ

Model	AUC (95% CI)	Sensitivity (95% CI)	Specificity (95% CI)	Youden	Cut-off
SVM	0.733 (0.718–0.748)	0.709 (0.694–0.724)	0.711 (0.696–0.726)	0.421	0.166
NB	0.762 (0.747–0.777)	0.621 (0.606–0.636)	0.783 (0.768–0.798)	0.405	0.011
RF	0.725 (0.710–0.740)	0.679 (0.664–0.694)	0.690 (0.675–0.705)	0.370	0.229
KNN	0.715 (0.700–0.730)	0.801 (0.786–0.816)	0.556 (0.541–0.571)	0.357	0.4
LR	0.775 (0.760–0.790)	0.781 (0.765–0.797)	0.670 (0.655–0.685)	0.452	0.250
AdaBoost	0.783 (0.770–0.796)	0.665(0.652–0.678)	0.824 (0.811–0.837)	0.490	0.492
XgBoost	0.785 (0.770–0.800	0.828 (0.813–0.843)	0.671 (0.656–0.686)	0.499	0.302
LightGBM	0.796 (0.781–0.811)	0.812 (0.797–0.827)	0.709 (0.694–0.724)	0.521	0.281
CatBoost	0.801 (0.786–0.816)	0.818 (0.803–0.833)	0.711 (0.696–0.726)	0.528	0.248

AdaBoost – adaptive boosting, AUC – area under curve, CaatBoost – categorical boosting, CI – confidence interval, KNN – K-nearest neighbours, LightGBM – light gradient boosting machine, LR – ogistic rRegression, NB – naïve Bayes, SVM – support vector machine, XGBoost – extreme gradient boosting

## DISCUSSION

As far as we know, this is the first study to develop a COPD screening questionnaire designated for the Chinese population, with its development directly guided by ML algorithms. To do this, we first established a primary version of the COPD-EQ questionnaire with nine items through using the Delphi approach to achieve consensus among Chinese COPD experts, after which we evaluated its validity and reliability on a sample of 1824 participants from 12 centres. By item score assignments assisted by the CatBoost model and SHAP method, we selected six out of nine items in the primary questionnaire to form the final version of the COPD-EQ. Applying the scoring-based method similar to the COPD-PS, the COPD-EQ questionnaire achieved a significantly higher AUC of 0.734 than the COPD-PS, demonstrating its screening ability.

Six items included in the final questionnaire cover the topics of age, cigarette smoking history and amount, biomass exposure history, dyspnoea frequency, and cough symptoms. Our study proved that age was a significant risk factor for COPD, which is consistent with previous research [3,9,27]. In China, the incidence of COPD increases with age, with the highest morbidity in the population aged above 70 years [3]. Kim et al. [27] found that annual FEV1 decline significantly accelerated among COPD patients in an older age.

Cigarette smoke exposure is another risk factor for the decline of lung function and the incidence of COPD [28]. Similarly, having a smoking history was conducive to accurately screening COPD patients in our study. A meta-analysis involving 24 studies in the Chinese population confirmed that smokers had a significantly higher risk of COPD than non-smokers [29]. A recent study from the US showed that the prevalence of chronic obstructive pulmonary disease was 15.2% among current smokers, 7.6% among former smokers, and 2.8% among non-smokers after adjusting for age and other confounding factors [30]. Moreover, COPD can also be attributable to biomass exposure, especially in women patients [31]. Biomass fuel burning, including coal, wood, and crops, can generate air particulate matter and lead to toxicant exposure in household members [32].

Dyspnoea is a major sign and influencing factor of restrictive living activities in COPD patients [33]. Besides, Gruenberger and colleagues [34] reported that greater dyspnoea corresponded to lower health-related quality of life among European COPD patients. Chronic cough is also a central, definitional symptom of COPD [35]. Interestingly, it was proved that the presence of chronic cough/phlegm was an independent risk sign to identify subjects with a high risk of developing COPD in young adults aged 20 to 44 years [36]. A recent survey from Japan reported that screening subjects with chronic cough through a comprehensive physical examination combined with a further detailed examination might be an effective alternative for better managing chronic cough [37]. Furthermore, higher levels of cough in COPD patients were associated with worse clinical and quality-of-life outcomes [38].

The COPD-EQ questionnaire developed from our study may be more effective for COPD than COPD-PS, as shown by a higher AUC. Currently, COPD-PS is the most widely used COPD screening questionnaire and has been validated and applied around the world [39]. It contains five items spanning four relevant topics (i.e. dyspnoea, cough, cigarette smoke history, age) and one distinct topic (i.e. physical activity), compared to the COPD-EQ. The number of items in COPD-EQ is comparable to or below that of existing questionnaires, which have between five to eight items [12–15,18]. This highlights its advantage in terms of cost-effectiveness in screening COPD patients. However, it also has several other positive novelties that address the limitations of previous questionnaires: the integration of expert consensus via the Delphi method; the validation with a large and diverse population; the application of ML for optimisation; and the focus on key predictive items. According to the Youden index, 4.0 was determined as the best threshold for the COPD-EQ questionnaire, and the corresponding sensitivity and specificity were 67.58% and 79.20%. In real practice, the choice of threshold should refer to regions and accessibility of medical resources.

ML methods have been widely used in many medical fields such as personalised treatment, drug discovery, prediction of disease progression, and early cancer screening in recent years. Because of their ability to find connections and identify patterns from big data, they have also been shown to improve the accuracy and reliability of diagnostic systems for many diseases. In the final phase of this study, we developed a series of ML models to further enhance the screening performance of COPD-EQ. In all models, the CatBoost model achieved the highest AUC score of 0.801, which is a significant improvement over the traditional scoring-based method on COPD-EQ, suggesting that ML models can improve the questionnaire’s screening ability. There is, however, a need for a further large-scale study to verify the feasibility and effectiveness of the ML-based screening method and confirm whether we could use it in clinical practice.

Some limitations need to be acknowledged in this study. First, enrolling a different population as the testing cohort may better illustrate the validity of the COPD-EQ questionnaire. Second, translation and validation for COPD-EQ in more countries are expected in the future. Third, we may have had issues with recall bias, which occurs when participants do not accurately remember past events or exposures, thereby leading to misclassification and biased results. To minimise this, we designed the COPD-EQ questionnaire to include clear and specific questions. Additionally, we compared self-reported data with clinical records where possible.

## CONCLUSIONS

In this work, we successfully established an effective and convenient screening questionnaire for COPD, the COPD-EQ. In the first phase, we implemented a Delphi method to form the primary nine-item version of COPD-EQ. In the second phase, we conducted a nationwide multi-centre study to validate and refine the COPD-EQ. Based on the data set collected in the study, we first develop a scoring assignment for COPD-EQ with the help of ML models and the SHAP method. The optimal scoring scheme discarded three question items and resulted in a briefer questionnaire. Finally, we developed a series of ML models which used the responses of the subjects as features. The CatBoost model achieved the highest average AUC scores in cross-validation and outperformed the traditional scoring-based screening method. The resulting novel COPD-EQ questionnaire can recognise subjects with high risks of COPD and can be applied as a practical COPD screening tool in clinical practice.

## Additional material


Online Supplementary Document
