# Supplementary material for: Machine learning-assisted construction of COPD self-evaluation questionnaire (COPD-EQ): a national multicentre study in China
Source: J Glob Health. 2025 Jan 3;15:04052. doi: 10.7189/jogh.15.04052 (PMC11699521; doi:10.7189/jogh.15.04052)
Supplement: Online Supplementary Document [file jogh-15-04052-s001.pdf]

Supplement to: Ma Y, Zhan Z, Chen YH, Zhang J, Li W, He Z, Xie J, Zhao H, Xu A, Peng K, Wang G, Zeng Q, Yang T, Chen Y, Wang C. Machine learning-assisted construction of COPD self-evaluation questionnaire (COPD-EQ): a national multicentre study in China. J Glob Health. 2025;15:04052.

Appendix Table 1 The primary 9-item version of COPD-EQ questionnaire

Items question	Response Choices
How old are you?	☐ Aged 35-49<br>☐ Aged 50-59<br>☐ Aged 60-69<br>☐ Aged 70+
How many cigarettes have you smoked?<br>(Pack-years)	☐ 0<br>☐ 0 – 10<br>☐ 10 – 20<br>☐ >20
Do you have exposed to biomass smoke for more than half a year?	☐ No<br>☐ Yes
During the past year, how much of time did you feel short of breath?	☐ None of the time<br>☐ A little of the time<br>☐ Some of the time<br>☐ Most of the time<br>☐ All the time
When do you have dyspnea?	☐ No dyspnea<br>☐ During strenuous activities<br>☐ During walking quickly on the flat ground or climbing a small slope<br>☐ Slower than peers when walking on the flat ground, or needs to rest<br>☐ Severe dyspnea leads to inability to leave home, or dyspnea when wearing and undressing
Do you often cough when you don't have a cold?	☐ No<br>☐ Yes
Have you ever coughed up something, such as mucus or sputum?	☐ Never<br>☐ Yes, cough occasionally when having a cold or chest infection<br>☐ Yes, cough for a few days every month<br>☐ Yes, cough for most days<br>☐ Yes, cough every day
Did you suffer from chronic respiratory disease as a child?	☐ No<br>☐ Yes
Whether your first-degree relatives (parents, children, siblings) have a history of respiratory disease (such as chronic bronchitis, emphysema, asthma)?	☐ No<br>☐ Yes

Appendix Table 2 The final 6-item version of COPD-EQ questionnaire

Items	Responses	Score
How old are you?	☐ Aged 35-49	0
	☐ Aged 50-59	1
	☐ Aged 60-69	1.5
	☐ Aged 70+	3
How many cigarettes have you smoked? (Pack-years)	☐ 0	0
	☐ 0 – 10	1.5
	☐ 10 – 20	1.5
	☐ >20	1.5
Do you have exposed to biomass smoke for more than half a year?	☐ No	0
	☐ Yes	0.5
During the past year, how much of time did you feel short of breath?	☐ None of the time	0
	☐ A little of the time	0.5
	☐ Some of the time	0.5
	☐ Most of the time	1.5
	☐ All the time	2
When do you have dyspnea?	☐ No dyspnea	0
	☐ During strenuous activities	0.5
	☐ During walking quickly on the flat ground or climbing a small slope	0.5
	☐ Slower than peers when walking on the flat ground, or needs to rest	0.5
	☐ Severe dyspnea leads to inability to leave home, or dyspnea when wearing and undressing	1
Have you ever coughed up something, such as mucus or sputum?	☐ Never	0
	☐ Yes, cough occasionally when having a cold or chest infection	1
	☐ Yes, cough for a few days every month	1
	☐ Yes, cough for most days	1
	☐ Yes, cough every day	1
